# Characterizing Sources of Small DC Motor Noise and Vibration

**DOI:** 10.3390/mi9020084

**Published:** 2018-02-15

**Authors:** Yong Thung Cho

**Affiliations:** Division of Mechanical and Automotive Engineering, Kongju National University, ChunAn, ChoongNam 31080, Korea; cho.yong@gmail.com; Tel.: +82-41-521-9277

**Keywords:** motor noise, micro motor, sound visualization, noise source identification

## Abstract

Small direct current (DC) motors are widely used due to their low cost and compact structure. Small DC motors of various designs are available on the market in different sizes. The smaller the motor, the more closely it may be used by individuals. Contrary to the size and simplicity of these motors in terms of structural design, sources of motor noise and vibration can be quite diverse and complicated. In this study, the source of motor noise and vibration was visualized over a very wide range of frequencies. The particle velocity of the motor was reconstructed from nearfield sound pressure measurements of motor noise. In addition to noncontact measurements conducted on a motor running at constant speed, the particle velocity of a stationary motor due to the impulse of an impact hammer was measured with an accelerometer. Furthermore, motor noise was measured under motor run-up conditions with different rotational speeds. As a result, by combination of these three methods, the sources of motor noise were accurately identified over a wide range of frequencies.

## 1. Introduction

Small direct current (DC) motors are widely used in toys, automobiles, and personal appliances due to their low cost and compact structure; they typically consist of housing, permanent magnets (stator), armature (rotor), brushes, etc. Various designs for small DC motors are available on the market, with different sizes and numbers of poles. The smaller the motor, the more closely it may be used by individuals. Contrary to the size and simplicity of these motors in terms of structural design, sources of motor noise and vibration are often complicated, tracing back to electro-magnetic forces between armature and permanent magnets or the switching of brushes, housing resonances, bearings, etc., which are quite diverse. Electro-magnetic force and cogging torque are major characteristics of such motors, and these aspects have been actively analyzed [1,2,3]. Measurement and analytical work related to motor noise sources is relatively few, possibly due to the diversity of motor types and complexity of noise sources. Noise radiated from brushless DC motors has been measured, and resonance from structural and acoustical excitation has been shown in addition to simulation matching [4].

The typical dimensions of a micro motor shown in present work are roughly 30 mm in both diameter and length. The frequency of sound radiation from these structures ranges from 80 Hz to higher than 8 kHz. Due to their small size, only two or three times larger than a typical transducer, accurately identifying the source of small motor noise is challenging. Acoustical holography has been implemented to visualize the source of motor noise and vibration overall over a very wide range of frequencies by using a relatively small number of microphones. Since the particle velocity of the source surface represents the behavior of a source more accurately than sound pressure on the measurement surface, the particle velocity of a motor was reconstructed from nearfield sound pressure measurements of motor noise. In addition to noncontact measurements conducted on a motor running at constant speed, the particle velocity of a stationary motor due to the impulse of an impact hammer on the motor housing was measured with an accelerometer. Furthermore, motor noise was measured under motor run-up conditions with different rotational speeds by varying the motor input voltage, which was useful for characterizing the noise directly related to rotational forces and structural or acoustical resonances.

## 2. Measurement and Sound Visualization

An acoustical holography procedure [5], which also could be described as an inverse system procedure, was implemented to visualize motor noise. Acoustical holography was first introduced for projection of the measurement in spherical coordinates [6]. A version using cylindrical coordinates was later introduced to make measurement surfaces conform more closely to source geometry [7]. An alternative holography procedure, statistically optimized near-field acoustical holography (SONAH), was derived to reduce spatial truncation and the size of the measurement surface [8,9]. SONAH was also modified for projection of measurement in cylindrical coordinates [10]. Cylindrical SONAH was implemented to visualize noise radiated from power seat slide motors [11]. By using fixed reference signals during measurement, a scan can be completed with a relatively small number of microphones for successful acoustical holography reconstruction [12,13]. In the present work, three different types of measurements, cylindrical SONAH, structural impulse using an impact hammer, and a motor run-up test were matched to identify noise sources in a small DC motor.

The sound pressure on a cylindrical surface of radius *r* can be expressed as

(1) $$p(r,\phi ,z)=\sum_{m=-\infty }^{m=\infty }\frac{1}{2\pi }\int_{-\infty }^{\infty }P_{m}(r,k_{z})e^{im\phi }e^{ik_{z}z}dk_{z}$$

where *P_m_*(*r*,*k*_z_) is the cylindrical wave number spectrum of *p_m_*(*r*,*φ*,*z*) for the *m*th circumferential component of the sound field, and *k_z_* is the axial component of the wave number. The wave number spectrum at radius *r* can also be expressed in terms of the wave number spectrum of the sound field on a cylindrical source surface of radius *r_s_*:

(2) $$P_{m}(r,k_{z})=\frac{H_{m}^{\left(1\right)}(k_{r}r)}{H_{m}^{\left(1\right)}(k_{r}r_{s})}P_{m}(r_{s},k_{z})$$

where $H_{m}^{\left(1\right)}$ is the *m*th order Hankel function and the radial wave number is

(3) $$k_{r}=[\begin{array}{l} \sqrt{k^{2}-k_{z}{}^{2}}\;\mathrm{for}\;\left|k\right|\geq \left|k_{z}\right|\\ i\sqrt{k_{z}{}^{2}-k^{2}}\;\mathrm{for}\;\left|k\right|<\left|k_{z}\right| \end{array}$$

with *k* = *ω*/*c*, *ω* being the angular frequency and *c* the ambient sound speed. The spatial distribution of the sound pressure at radius *r* can then be found by inverse transformation of the projected wave number spectrum at *r*:

(4) $$p(r,\phi ,z)=\sum_{m=-\infty }^{m=\infty }\frac{1}{2\pi }\int_{-\infty }^{\infty }\frac{H_{m}^{\left(1\right)}(k_{r}r)}{H_{m}^{\left(1\right)}(k_{r}r_{s})}P_{m}(r_{s},k_{z})e^{im\phi }e^{ik_{z}z}dk_{z}.$$

Now, define a three-dimensional cylindrical wave function, $\Phi_{k_{z},m}(r,\phi ,z)$, as

(5) $$\Phi_{k_{z},m}(r)=\Phi_{k_{z},m}(r,\phi ,z)\equiv \frac{H_{m}^{\left(1\right)}(k_{r}r)}{H_{m}^{\left(1\right)}(k_{r}r_{s})}e^{im\phi }e^{ik_{z}z},\;r\geq r_{s}.$$

The sound pressure on the cylindrical surface at radius *r* can then be expressed using the wave function, $\Phi_{k_{z},m}(r,\phi ,z)$, as

(6) $$p(r)=\frac{1}{2\pi }\sum_{m=-\infty }^{m=\infty }\int_{-\infty }^{\infty }P_{m}(r_{s},\;k_{z})\Phi_{k_{z},m}(r)dk_{z}.$$

When **r***_h_* is used to represent positions on the measurement (or hologram) surface at *r* = *r_h_* > *r_s_*, an expression is obtained for the pressure on the hologram surface:

(7) $$p(r_{h})=\frac{1}{2\pi }\sum_{m=-\infty }^{m=\infty }\int_{-\infty }^{\infty }P_{m}(r_{s},\;k_{z})\Phi_{k_{z},m}(r_{h})dk_{z}.$$

Assume that sound pressure *p*(**r**) at an arbitrary position is represented as a linear combination of the measured sound pressure data, $p(r_{h,j})$:

(8) $$p(r)\approx \sum_{j=1}^{J}c_{j}(r)\cdot p(r_{h,j}).$$

The same coefficients *c_j_* in Equation (8) also provide a good estimation for the cylindrical wave functions:

(9) $$\Phi_{k_{zq},m}(r)\approx \sum_{j=1}^{J}c_{j}(r)\Phi_{k_{zq},m}(r_{h,j}),\;m=1\;\ldots \;M,\;q=1\;\ldots \;N.$$

The various quantities involved in the calculation are defined in the form of matrices and vectors as

(10) $$A\equiv \left[\Phi_{k_{zq},m}(r_{h,j})\right],\;\alpha (r)\equiv \left[\Phi_{k_{zq},m}(r)\right],\;c(r)\equiv \left[c_{j}(r)\right].$$

Equation (9) can then be expressed in matrix form as

(11) α(r)≈Ac(r).

The regularized least squares solution for the weight vector, **c**(**r**), is then

(12) $$c(r)={\left(A^{+}A+\theta^{2}I\right)}^{-1}A^{+}\alpha (r),$$

where ^+^ denotes the Hermitian or conjugate transpose, **I** is the identity matrix, and the regularization parameter, *θ*, is

(13) $$\theta^{2}={\left[A^{+}A\right]}_{ii}10^{-SNR/10},$$

where *SNR*, the regularization parameter, depends on the signal-to-noise ratio of the measurement signal and the reconstruction location from the measurement. The subscript *ii* is used here to denote the diagonal elements of a matrix. The reconstructed pressure, *p*(**r**), is expressed as

(14) $$p(r)\approx \sum_{j=1}^{J}c_{j}(r)p(r_{h,j})=p^{T}(r_{h})c(r)=p^{T}(r_{h}){\left(A^{+}A+\theta^{2}I\right)}^{-1}A^{+}\alpha (r),$$

where **p**(**r***_h_*) is the vector of measured pressures.

The radial particle velocity on the reconstruction surface, *u_r_*(**r**), can be found using Euler’s equation:

(15) $$u_{r}(r)=\frac{1}{i\rho_{o}\omega }\frac{\partial p(r)}{\partial r}.$$

By substituting the estimated spatial distribution of the sound pressure from Equation (14) into Equation (15), the radial particle velocity, *u_r_*(**r**), is obtained as

(16) $$\begin{array}{ll} u_{r}(r) & \approx \frac{1}{i\rho_{o}\omega }\frac{\partial }{\partial r}\left[p^{T}(r_{h}){\left(A^{+}A+\theta^{2}I\right)}^{-1}A^{+}\alpha (r)\right]\\ & =p^{T}(r_{h}){\left(A^{+}A+\theta^{2}I\right)}^{-1}A^{+}\beta (r)=p^{T}(r_{h})d(r), \end{array}$$

where **d**(**r**) is the transfer matrix between measurement pressure and the reconstructed particle velocity, and the vector **β**(**r**) is defined as

(17) $$\beta (r)\equiv \frac{1}{i\rho_{o}\omega }\frac{\partial \alpha (r)}{\partial r}=\left[\frac{1}{i\rho_{o}\omega }\frac{\partial }{\partial r}\Phi_{k_{zq},m}(r)\right].$$

The elements of **β**(**r**) are modified cylindrical wave functions, which by use of Equation (5) can be expressed as

(18) $$\Phi_{k_{zq},m}^{u}(r,\phi ,z)\equiv \frac{-ik_{r}}{\rho_{o}\omega }\frac{H_{m}^{{\left(1\right)}^{\prime }}(k_{r}r)}{H_{m}^{\left(1\right)}(k_{r}r_{s})}e^{im\phi }e^{ik_{zq}z},$$

where the superscript prime denotes differentiation with respect to the function’s argument and *ρ*_o_ is the ambient density. The vector **β**(**r**) can then be written as

(19) $$\beta (r)\equiv \left[\Phi_{k_{zq},m}^{u}(r)\right].$$

Now, pressure and particle velocity can be reconstructed at other surfaces, such as source surfaces, from the measurement pressure. So, more detailed and accurate information about the source can be obtained using the reconstructed properties than with measurement pressure alone.

Particle velocity of the motor housing due to impulsive force of impact hammer can be measured directly using the accelerometer for undamped systems or system with small damping. Unit impulse or the delta function, *δ*(*t*), is defined as [14,15]

(20) δ(t−ξ)=0,

for all *t*, except *t* = *ξ* and delta function becomes very large or infinite when *t* = *ξ*, and its integral becomes unity, which is

(21) $$\int_{0}^{\infty }\delta (t-\xi )dt=1,$$

for positive *ξ*. So, the time integral of delta function multiplied by function *f*(*t*) can be evaluated as

(22) $$\int_{0}^{\infty }f(t)\delta (t-\xi )dt=f(\xi ).$$

From the equation of motion of a point mass with external force, *F*, applied,

(23) Fdt=mdv,

impulse is represented as,

(24) $$\hat{F}=F\Delta t=m\Delta v=mv.$$

When the mass is initially at rest, *v* is the velocity of mass after impulse. Undamped free vibration solution of a spring-mass system initially located at origin is [15]

(25) $$x=\frac{\dot{x}(0)}{\omega_{n}}\sin\omega_{n}t,$$

where *x* is the displacement. Since velocity of mass after impulse, *v*, is the initial velocity, the displacement *x* can be represented by using the impulse and mass, which is

(26) x=vωnsinωnt=F^mωnsinωnt=−F^mωnIm(e−jωnt).

By differentiation of the displacement in time, and relationship between the velocity and the acceleration is reduced to

(27) $$\dot{x}=-\frac{1}{j\omega_{n}}\ddot{x}.$$

As a result, the particle velocity of undamped or small damping systems can be estimated from the measurement of the acceleration of impulse excitation.

## 3. Measurement Description

Sound pressure of a motor rotating at constant speed was measured on a cylindrical surface to reconstruct the particle velocity of a source surface. In addition, the particle velocity of stationary motor housing due to an impulse by an impact hammer on the housing was measured with an accelerometer. Also, motor noise was measured under motor run-up conditions with different rotational speeds by varying the motor input voltage.

**Figure 1:**
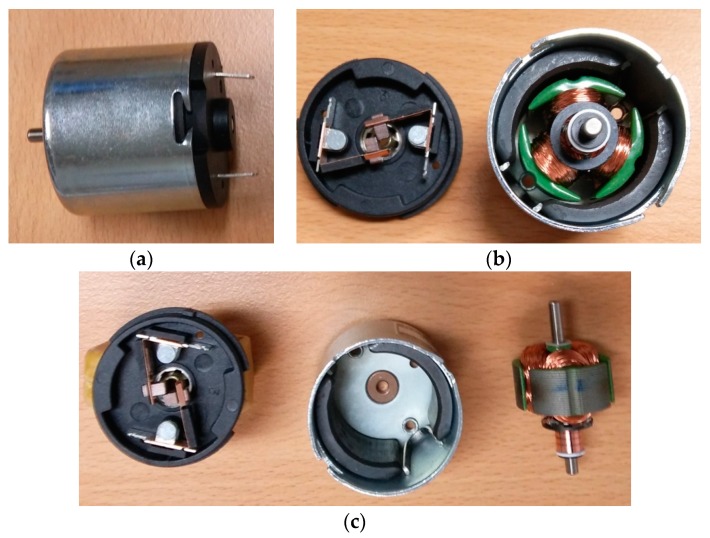
The exterior and interior of a small direct current (DC) motor with brushes used for noise measurement: (**a**) small DC motor exterior; (**b**) three-pole rotor installed inside the motor housing with two permanent magnets and the brush cap open; (**c**) three-pole rotor with grooved armature separated from the motor housing.

The exterior and interior of a small DC motor with brushes used for noise measurements are shown in Figure 1. The exterior diameters of 12 V DC motor housing and armature were 34 mm and 24 mm, respectively. The lengths of the motor housing and armature were 30 mm and 10 mm, respectively. Two brushes were located at the bottom cap as shown in Figure 1b. In the present work, the location of the brushes is referred to as the bottom, and the location of the shaft is referred to as the top. The two permanent magnets were restrained inside the motor housing, and the rotor consisted of armature with three poles. Three ventilation holes on the top surface of the motor housing are shown in Figure 1b,c.

**Figure 2:**
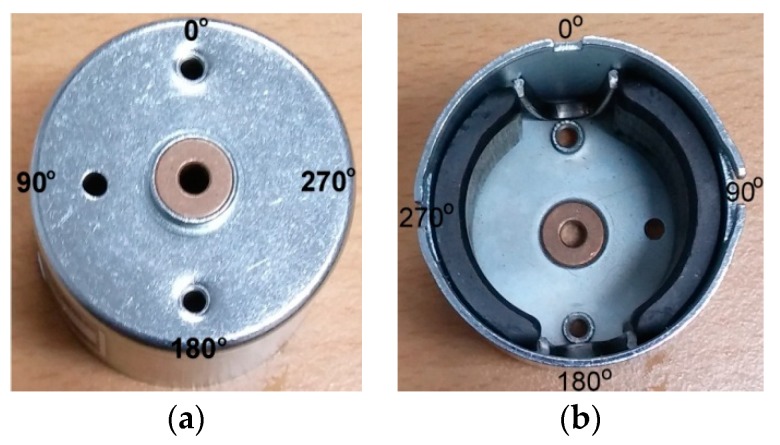
The exterior and interior of small DC motor housing with angular coordinates used for noise measurement: (**a**) exterior of the motor housing, top view; (**b**) interior of the motor housing, bottom view.

**Figure 3:**
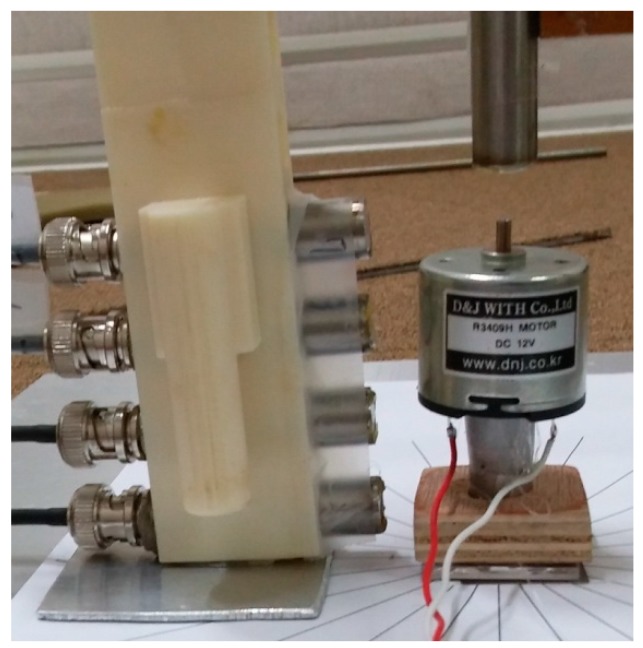
A small DC motor and measurement microphones positioned in the vicinity of the motor. A stationary reference microphone positioned above the center of the motor shaft, and scanning microphones located in parallel to the axial direction of the motor.

Also, the armature was grooved to prevent the ventilation holes from being closed while the motor was rotating. The exterior and interior of a small DC motor with angular coordinates used for noise measurement are shown in Figure 2. The locations of three ventilation holes are clearly shown in Figure 2, at 0°, 90°, and 180°. The small DC motor and microphones positioned for measurement are shown in Figure 3. A stationary reference microphone was also positioned above the center of the motor shaft, and scanning microphones were located in parallel to the axial direction of the motor, as shown in Figure 3. The scanning microphone position shown in Figure 3 represents the 0° mark, which is consistent with the coordinates shown in Figure 2. The scanning microphones were rotated counter-clockwise to obtain measurements in increments of 15°. The total number of measurements in the circumferential direction was twenty-four. One stationary reference microphone was located on the top of the motor shaft, and four scanning microphones were located with 2 cm spacing in the axial direction. Relatively few microphones were used for measurement since the motor was relatively small. However, the entire cylindrical source surface around the motor was measured.

Accelerometer model PCB 353B15 (PCB Piezotronics, Inc., Depew, NY, USA) and impact hammer model PCB 086C03 (PCB Piezotronics, Inc., Depew, NY, USA) were used for motor housing acceleration measurement. The location of impact was at the 0° mark on the motor housing center, and measurements were taken at 90° and 180° on the motor housing center relative to the location of impact. Output from the accelerometer and impact hammer was gathered via the input of a signal conditioner model PCB 482C (PCB Piezotronics, Inc., Depew, NY, USA). Output from the signal conditioner was directed as analog input to a data acquisition model NI-USB-6363 (National Instruments Corporation, Austin, TX, USA). Finally, output from the NI-USB-6363 was sent to a laptop computer, model Acer Aspire 5736Z-4801 (Acer Inc., New Taipei City, Taiwan), and the measurement data was saved on the laptop.

A total of five microphones, including four scanning and one fixed microphones as shown in Figure 3, were used for motor sound pressure measurements. The entire cylindrical surface was scanned while the motor was running at constant speed at 12 V. For motor run-up operation from 6 V to 15 V, the position shown in Figure 3 represents the 0° mark. For run-up operation of the motor, sound pressure was measured only at 0°. The microphones were calibrated at 1000 Hz, 94 dB, and connected to a microphone amplifier. Output from the microphone amplifier was directed to the NI-USB-6363, which was connected to a laptop computer, and the measurement data was saved on the laptop.

All signals in the present work were sampled at 44.1 kHz. The Hann window was applied to measurements from the microphones. A uniform window was applied to signals from the accelerometer and impact hammer. Microphone spacing in the axial direction was 2 cm. The radius of measurement was 2.7 cm, which was 1 cm from the motor housing surface. The angular increment of measurement was 15°, and the corresponding measurement spacing in the angular direction was about 0.707 cm. Due to the relatively small radius of the motor and measurement surface, measurement spacing in the angular direction was smaller than in the axial direction.

It would be ideal to take all measurements in free boundary condition. However, for convenience, all measurements of sound pressure and acceleration of the motor shown in the present work were taken with the motor located on a relatively rigid boundary, a 20 mm thick aluminum plate. 

## 4. Motor Excitation Forces

Electro-magnetic force defines one major characteristic of a motor and is closely related to motor noise and vibration; it has been actively analyzed [1,2,3,4,5]. Any periodic function can be represented as the sum of harmonic functions of multiples of fundamental frequency [16]. Moreover, a periodic function with fundamental frequency, *f*_f_, can be decomposed to a harmonic function of multiples of fundamental frequencies, as

*f*_p_ = *mf*_f_, (28)

where *f*_p_ is the frequency of the periodic function and *m* is a positive integer.

Unbalanced mass or forces of armature, possibly due to geometric non-symmetry during the manufacturing process, are also a potential cause of motor noise and vibration. The frequency of excitation due to an unbalanced force of armature, *f*_u_, is

*f*_u_ = *mf*_r_, (29)

where *f*_r_ is motor rotational speed in revolutions per second. For *m* = 1, *f*_u_ is the fundamental frequency of motor rotation. The frequency of excitation due to electro-magnetic force by armature at a point on the housing, *f*_e_, is

*f*_e_ = *mpf*_r_, (30)

where *p* is the number of poles of armature, which is three for the motor shown in the present work. However, the frequency of the entire housing, *f*_h_, is

*f*_h_ = *mL*_cm__h_*f*_r_, (31)

where *L*_cmh_ is the least common multiple of the number of poles in armature and housing, which is six for the motor shown in present work.

The frequency of excitation due to a ventilation fan, *f*_v_, is

*f*_v_ = *mqf*_r_, (32)

where *q* is the number of blades in the ventilation fan. However, there was no ventilation or cooling fan in the motor used here. The frequency of excitation from forces from the brushes due to switching in the commutator, *f*_b_, is

*f*_b_ = *mL*_cm__b_*f*_r_, (33)

where *L*_cm__b_ is the least common multiple of the number of poles and brushes, which is six for the motor shown in the present work. The major forcing frequencies of the excitation are summarized in Table 1.

## 5. Measurement Results

Motor noise was measured under motor run-up conditions with different rotational speeds by varying the motor input voltage, which was useful for characterizing the noise directly related to rotational forces and structural or acoustical resonances. Also, the response of a stationary motor housing surface due to an impulse from an impact hammer on the motor housing was measured with an accelerometer. The sound pressure of a motor rotating at constant speed was also measured on a cylindrical surface to reconstruct the particle velocity of a source surface. 

**Figure 4:**
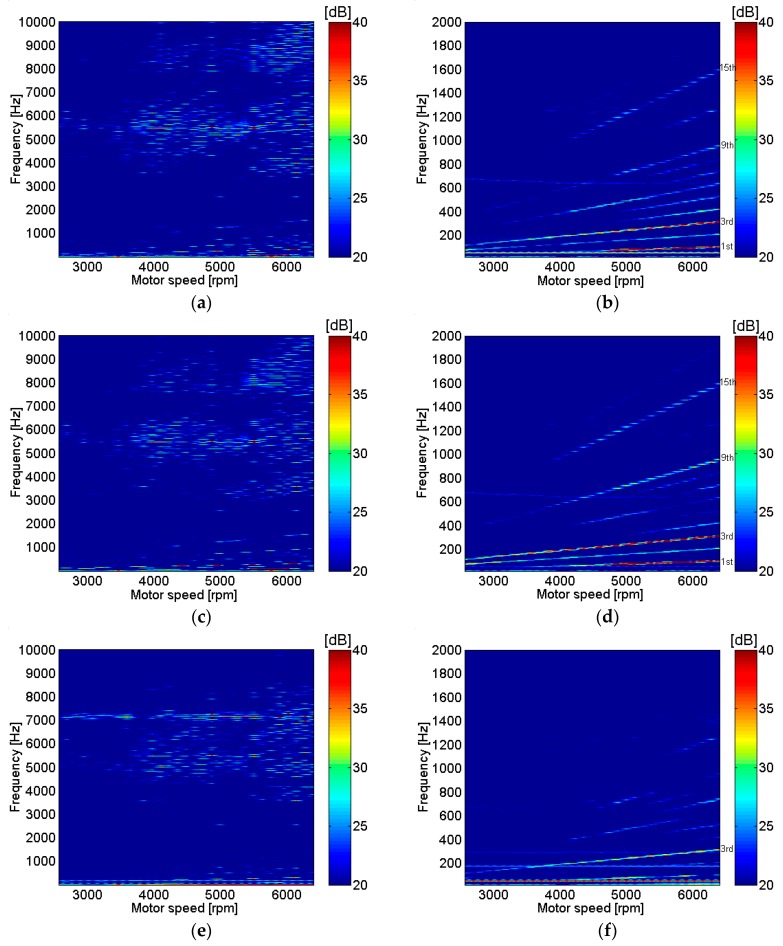
Motor noise measurement at different locations for motor run-up operation from 6 to 15 V with aluminum base plate support: (**a**) bottom end cap; (**b**) bottom end cap; (**c**) motor housing center; (**d**) motor housing center; (**e**) motor shaft center top; (**f**) motor shaft center top.

Motor noise without load was measured at different rotational speeds by varying the motor input voltage as shown in Figure 4. The motor input voltage was increased from 6 V to 15 V with 0.3 V increments, where the nominal motor input voltage was 12 V. The location of the microphones was at the 0° mark, as shown in Figure 2 and Figure 3. The results of measurements at different locations, such as the bottom end cap, housing center, and shaft center at the top, with different upper frequency ranges of 10 kHz and 2 kHz are shown in Figure 4. No weighting or linear weighting on the decibel scale were applied to the measurement results shown in Figure 4. The measurements from three different locations were relatively similar especially around 5000 Hz and the third motor rotating speeds. However, the measurement results at the motor housing center were the clearest compared to the results from other locations, as shown in Figure 4. Higher peaks were observed around 5000 Hz over a wide range of motor rotating speeds, especially at higher rotating speeds, which indicates that a major source of noise may be motor housing resonance around 5000 Hz, which is shown clearly in Figure 4c. In contrast, based only on motor run-up measurements, strong peaks near the first and third order of motor rotating speeds are clearly shown in Figure 4d, possibly due to unbalanced mass during rotation and electromagnetic forces between the armature and permanent magnets. Resonance around 7000 Hz was very clearly shown from the measurement on the top of the housing at motor shaft center, as shown in Figure 4e, which implied that the resonance was related to the top part of the motor. Sources of noise were also confirmed and identified by measurement of the particle velocity of the motor housing surface due to an impulse from an impact hammer and reconstruction of particle velocity of the motor housing surface from sound pressure measurements.

**Figure 5:**
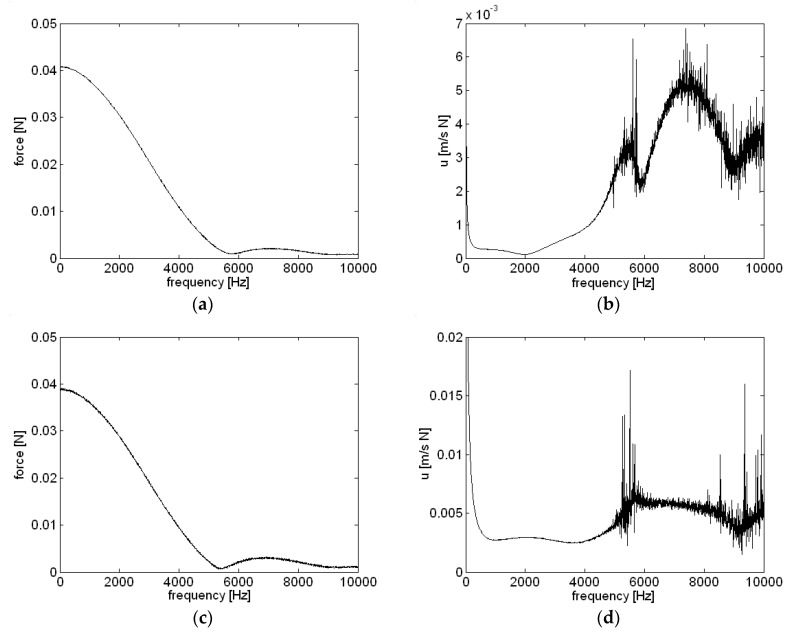
Response of the motor housing at different locations due to external excitation from a hammer: (**a**) 90°, force; (**b**) 90°, particle velocity; (**c**) 180°, force; (**d**) 180°, particle velocity.

The response of the motor housing surface at different locations due to an impulse from an impact hammer is shown in Figure 5. The motor housing was excited with an impulse from an impact hammer at 0° on the motor housing center, and measurements were taken at 90° and 180° with the same accelerometer, sequentially. The magnitude of the excitation force, the impulse from the impact hammer, is shown in Figure 5a,c. Even though a hammer with a steel tip was used to excite the housing at higher frequencies, the magnitude of the excitation force was relatively small at frequencies above 5000 Hz. However, the particle velocity measurements shown in Figure 5b,d indicate the possibility of structural resonance of housing at frequencies around 5000 Hz and 7000 Hz. Due to the fairly weak impulse, responses above 5000 Hz were very noisy both at 90° and 180°, but peak frequencies matched well with motor run-up measurements. The impact hammer used in present work, PCB 089C03 (PCB Piezotronics, Inc., Depew, NY, USA), was a general purpose impact hammer with frequency range of 8 kHz, and was readily available [16]. However, if the smaller impact hammer was used instead, possibly the higher input force and lower noise level in measured particle velocity in a high frequency region, especially above 4 kHz, should be shown.

The sound pressure of a motor rotating at constant speed with motor input voltage of 12 V was measured on a cylindrical surface to reconstruct the particle velocity of source surfaces. The microphones shown in Figure 3 were used to scan the sound pressure at twenty-four equally spaced locations in the circumferential direction on a cylindrical surface of radius 2.7 cm, which was 1 cm larger than the motor housing radius. Measurement increments in the axial direction were 2 cm, so as to avoid spatial aliasing in the axial direction the highest frequency was 8575 Hz, with the speed of sound in air being 343 m/s. Angular measurement increments were 15°, and measurement spacing in the angular direction was about 0.707 cm, so as to avoid spatial aliasing in the circumferential direction the highest frequency was 24257 Hz. As a result, 8575 Hz was considered the highest frequency to avoid spatial aliasing during reconstruction of particle velocity for measurements in the present work.

**Figure 6:**
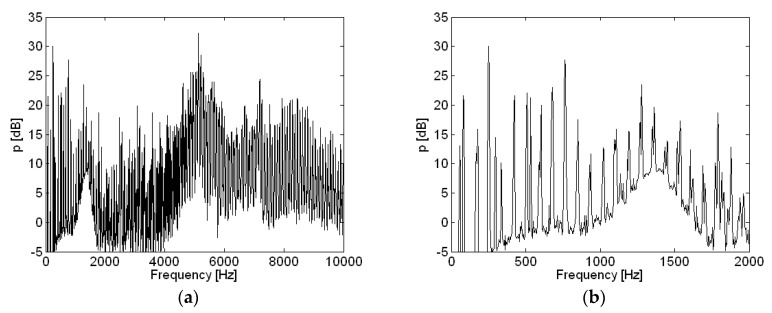
Spatially-averaged motor noise measurements for motor operation at 12 V with different frequency ranges: (**a**) 0–10000 Hz; (**b**) 0–2000 Hz.

**Figure 7:**
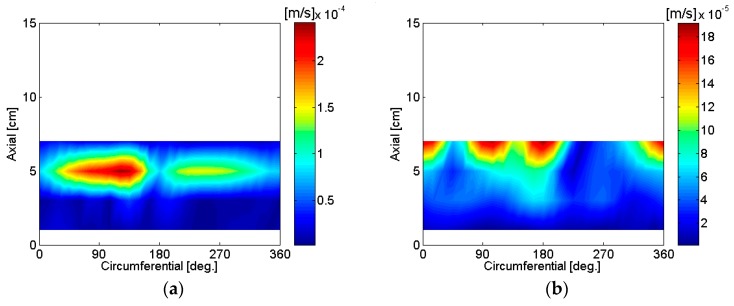

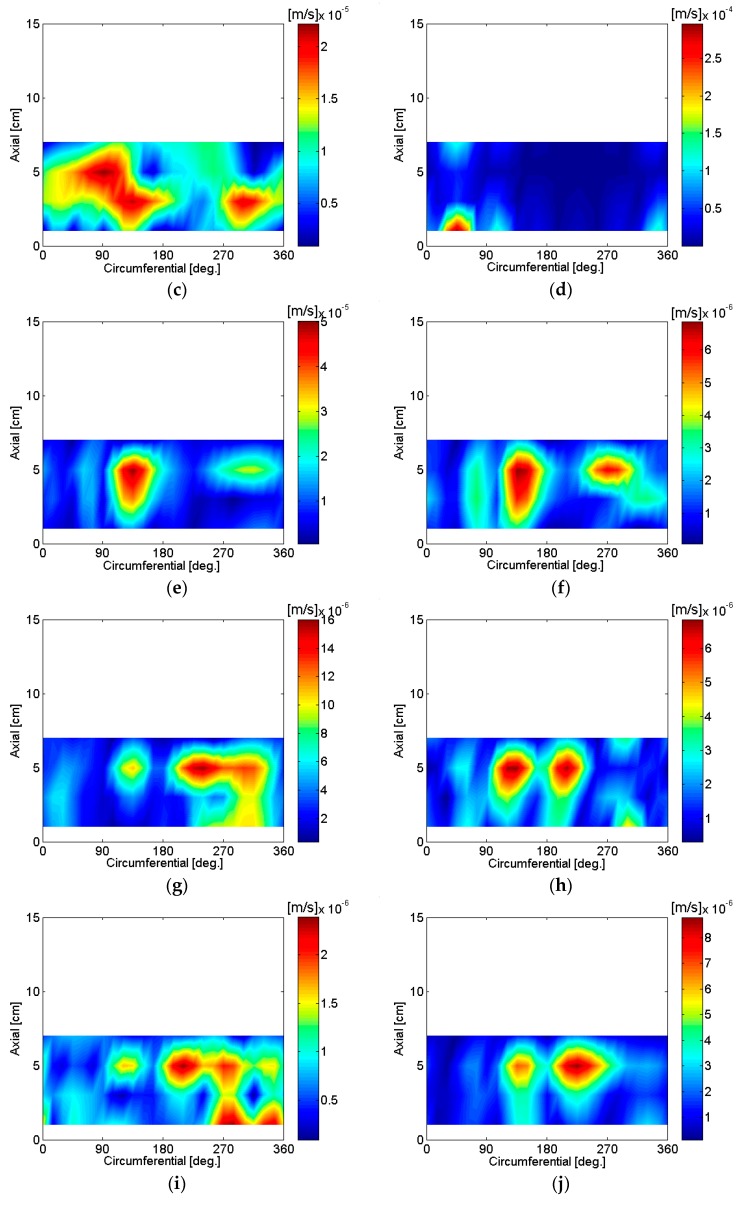

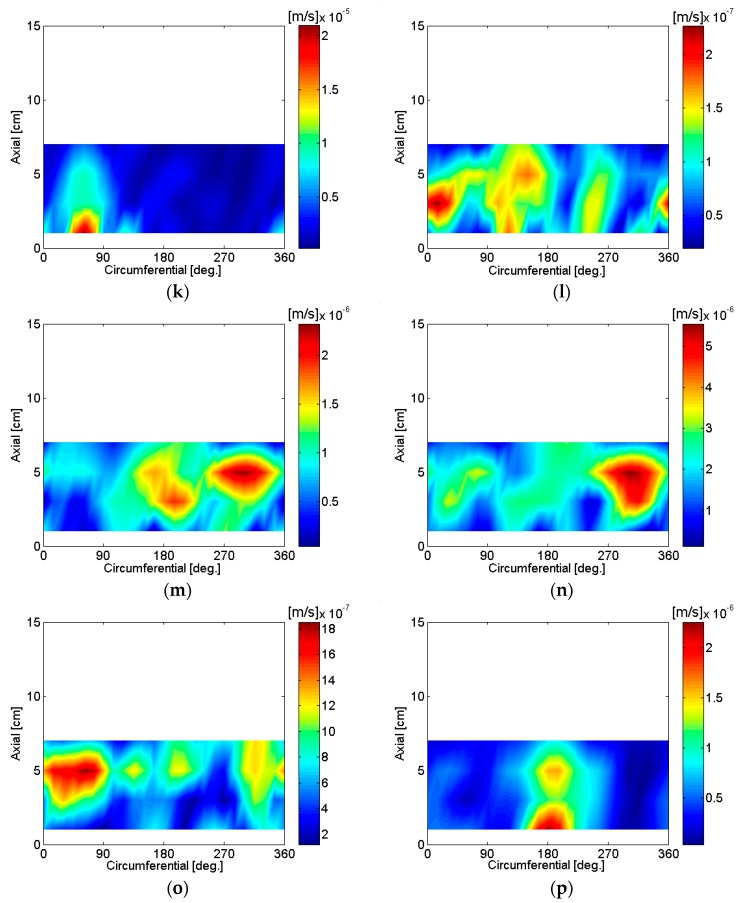
Reconstructed particle velocity of a motor supported on an aluminum base: (**a**) 84 Hz; (**b**) 252 Hz; (**c**) 508 Hz; (**d**) 532 Hz; (**e**)764 Hz; (**f**) 1024 Hz; (**g**) 1280 Hz; (**h**) 1360 Hz; (**i**) 1452 Hz; (**j**) 1792 Hz; (**k**) 3076 Hz; (**l**) 4680 Hz; (**m**) 4956 Hz; (**n**) 5124 Hz; (**o**) 7192 Hz; (**p**) 8456 Hz.

Spatially-averaged motor noise measurements for motor operation at 12 V are shown in Figure 6. The same results over a different frequency range are shown in Figure 6a,b. From the highest peaks of the spatially-averaged measurement pressure shown in Figure 6, sixteen frequencies were chosen, and the corresponding reconstructed source particle velocities are presented in Figure 7. All of the results shown in Figure 6 and Figure 7 are based on an A-weighted decibel level.

Based on the reconstructed source particle velocity results from the measurements shown in Figure 7, the motor was rotating at a speed of approximately 5120 revolutions per minute without load when 12 V were supplied. A description of the source particle velocity reconstruction for the motor is summarized and shown in Table 2.

Sound radiation from the motor housing due to an unbalanced force or mass at 84 Hz, which corresponds to a first-order rotation speed, is clearly shown in Figure 7a. Sound radiation from cooling holes on the top surface of the motor is clearly shown at 252 Hz in Figure 7b. The location of three holes is shown in Figure 2 and Figure 3. Even though there was no cooling fan inside the motor, 252 Hz corresponded to the first order of ventilation frequency considering the number of poles in the armature. Also, 252 Hz corresponded to the first-order electro-magnetic force excitation frequency. However, it is clearly shown in Figure 7b that the sound radiation from the three holes on the upper surface of the housing was more dominant than other sources of noise. Radiated sound due to the switching of brushes at 508 Hz is clearly shown in Figure 7c. The brushes were located on the bottom surface of the motor at 120° and 300°. A frequency of 508 Hz represented the first-order brush switching frequency, second-order electro-magnetic force excitation, and sixth-order motor rotation speed. Rather than sound radiation directly from the motor, reflection from the base support was dominant at 532 Hz. Similarly, reflection from the base support is clearly shown at 1452 Hz, 3076 Hz, and 8456 Hz. Sound radiated by electro-magnetic force excitation is shown at 764 Hz. In addition, sound radiated by electro-magnetic force excitation is shown at frequencies 1024 Hz, 1280 Hz, and 1792 Hz. The cause of the noise radiated at 1360 Hz may be due to the internal resonance rather than motor housing vibration. A relatively low response is shown in Figure 5b,d due to structural excitation of the motor housing, but a relatively high level of spatial-averaged sound pressure is shown in Figure 6 at 1360 Hz, which indicates motor internal resonance. One of the possibilities for the sound source at 1360 Hz is the internal acoustic resonance, but further investigation is required to confirm it. Radiated sound from the motor bottom cap is shown at 4680 Hz. Source of sound radiation at 4956 Hz is possibly due to coupled structural-acoustic resonances or acoustic resonances rather than by electro-magnetic force excitation, but further investigation is required to confirm it. 

Circular ring mode shape, *w*_3n_(*θ*), is represented as [17]

**Figure 8:**
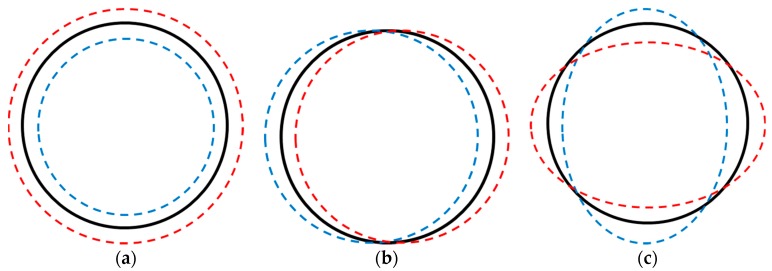
Circular ring mode shapes: (**a**) *n* = 0 mode; (**b**) *n* = 1 mode; (**c**) *n* = 2 mode.

*w*_3*n*_(*θ*) = *A*_n_cos[*n*(*θ* − Φ)], (34)

where *θ* is angle in circular direction, Φ is phase angle, and *n* is mode number. Circular ring mode shape is shown in Figure 8 for the cases of *n* = 0, 1 and 2.

Sound radiated by electro-magnetic force excitation and possibly a combination of circular ring *n* = 0 and *n* = 2 modes for the center and bottom part of the motor housing are shown at 5124 Hz in Figure 7h. For the circular ring mode shapes shown in Figure 8, the *n* = 2 mode shape consisted of four maximum magnitude values with opposite phase. Four peaks are shown at the bottom of the motor housing particle velocity reconstruction results at 5124 Hz, which indicate circular ring *n* = 2 mode at the open section of the motor housing. Similarly, sound radiated by electro-magnetic force excitation and circular ring *n* = 2 mode for the center of the motor housing are shown at 7192 Hz in Figure 7o. The response to structural excitation of the motor housing is shown in Figure 5b, and spatially-averaged sound pressure is shown in Figure 6a, which agrees very well with the particle velocity reconstruction results at both 5124 Hz and 7192 Hz. Even though there are some variations in motor run-up operation measurements depending on measurement location, measurements of motor run-up operation indicate a modal response from the motor housing at both 5124 Hz and 7192 Hz.

## 6. Conclusions

Measurement and analytical work related to motor noise sources are relatively few, possibly due to the diversity of motor types and complexity of noise sources. In the present work, noise radiated from a small DC motor was measured, and radiated noise due to electro-magnetic excitation and structural resonance by housing were clearly shown to match three different types of measurement results, verifying the accuracy of the procedures. Accurately identifying noise and vibration sources for small motors is challenging due to the small physical dimensions of these machines and their relatively wide frequency range. The range of frequencies for the motor shown in the present work was 84 Hz to 7192 Hz, and the highest frequency that avoided spatial aliasing of the measurements was 8575 Hz. Overall, major sources of motor noise and vibration were electro-magnetic forces, internal resonance, and motor housing resonance. Unbalanced forces on the rotor, top cooling holes on the motor housing, and the switching of brushes were also dominant sources of noise at 84 Hz, 252 Hz, and 508 Hz, respectively, which is clearly shown through the reconstructed particle velocity of source surfaces via measurement pressure.

Reconstruction of particle velocity on source surfaces via measurement pressure was very useful for identifying sources of noise in small motors over a wide range of frequencies. Noise radiation from the base support of the motor was accurately identified from the reconstruction. Also, spatially-averaged measurement pressure confirmed the results of the motor run-up test and the response to a structural impulse from an impact hammer. Noise radiated due to the rotating part of the motor and motor housing was clearly shown by the motor run-up test.

Motor run-up test is very useful for identification of sound radiation due to resonances and rotating parts of the motor. However, a relatively strong source, such as frequency of 1360 Hz, was not identified by using a run-up test due to its directivity. Results using only a small number of run-up test measurement locations may not be accurate, and enough number of measurement locations should be taken for accurate source identification.

Even though for the small motor shown in the present work, the source of noise is complicated, including such as structural resonance, acoustic resonance, and possibly coupled structural-acoustic resonance. Internal acoustic resonance was not identified using a run-up test with a relatively small number of measurement locations or impulse tests. However, internal acoustic resonance can be inferred from the reconstructed particle velocity on housing and frequencies of excitation. Further work is required to characterize internal resonance of motors in more detail.

It is difficult to verify measurement results accurately using only one type of measurement. However, by implementing three types of measurements simultaneously, sources of small motors can be identified more accurately.

## Figures and Tables

**Table 1:** Major forcing frequencies of the excitation.

**Number of Poles**	*p* = 3
	*q* = 3
	*L*_cmh_ = 6
	*L*_cmb_ = 6
**Forcing Frequencies**	*f*_u_ = *mf*_r_
	*f*_e_ = *mpf*_r_ = 3 *mf*_r_
	*f*_h_ = *mL*_cmh_*f*_r_ = 6 *mf*_r_
	*f*_v_ = *mqf*_r_ = 3 *mf*_r_
	*f*_b_ = *mL*_cmb_*f*_r_ = 6 *mf*_r_

**Table 2:** Description of source particle velocity reconstruction.

Frequency	Order	Description of Source
84 Hz	1st *f*_r_	Unbalanced force
252 Hz	3rd *f*_r_, 1st *f*_e_	Top ventilation
508 Hz	6th *f*_r_, 1st *f*_b_	Brush switching
532 Hz		Base reflection
764 Hz	9th *f*_r_, 3rd *f*_e_	Electro-magnetic force
1024 Hz	12th *f*_r_, 4th *f*_e_	Electro-magnetic force
1280 Hz	15th *f*_r_, 5th *f*_e_	Electro-magnetic force
1360 Hz	16th *f*_r_	Internal resonance
1452 Hz		Base reflection
1792 Hz	21st *f*_r_, 7th *f*_e_	Electro-magnetic force
3076 Hz	36th *f*_r_, 12th *f*_e_	Base reflection
4680 Hz		Bottom cap
4956 Hz	58th *f*_r_	Internal resonance
5124 Hz	60th *f*_r_, 20th *f*_e_	Housing bottom *n* = 2 mode
7192 Hz	84th *f*_r_, 28th *f*_e_	Housing center *n* = 2 mode
8456 Hz	99th *f*_r_, 33rd*f*_e_	Base reflection
